# The combined impact of dependency on caregivers, disability, and coping strategy on quality of life after ischemic stroke

**DOI:** 10.1186/s12955-018-1069-6

**Published:** 2019-02-07

**Authors:** Sarah Dewilde, Lieven Annemans, Andrew Lloyd, Andre Peeters, Dimitri Hemelsoet, Yves Vandermeeren, Philippe Desfontaines, Raf Brouns, Geert Vanhooren, Patrick Cras, Boudewijn Michielsens, Patricia Redondo, Vincent Thijs

**Affiliations:** 1 Department of Public Health, Faculty of Medicine, University of Ghent, Ghent, Belgium; 2 Services in Health Economics (SHE), Brussels, Belgium; 3 Interuniversity Centre for Health Economics Research, University of Ghent, Vrije Universiteit Brussel, Ghent, Brussels Belgium; 4 Bladon Associates, Oxford, UK; 5 Cliniques Universitaires Saint Luc, Brussels, Belgium; 6 Ghent University Hospital, Ghent, Belgium; 7 Université Catholique de Louvain, Yvoir, Belgium; 8 Centre Hospitalier Chrétien, Liège, Belgium; 9 Universitair Ziekenhuis Brussel, Brussels, Belgium; 10 Center for Neurosciences (C4N), Vrije Universiteit Brussel (VUB), Brussels, Belgium; 11 AZ Sint-Jan Brugge-Oostende AV, Bruges, Belgium; 12 Born Bunge Institute, University and University Hospital, Antwerp, Belgium; 13 Heilig Hart Ziekenhuis, Lier, Belgium; 14 CHU Ambroise Paré, Mons, Belgium; 15 Stroke Division, Florey Institute of Neuroscience and Mental Health, University of Melbourne, Melbourne, Australia; 16 Austin Health, Department of Neurology, Melbourne, Victoria Australia

**Keywords:** EQ-5D, Stroke, Disability, Dependency on caregivers, Coping, PRO, Utilities

## Abstract

**Background:**

To estimate the additional impact of coping and of being dependent on caregivers, over and above the large effects of disability on utility after ischemic stroke.

**Methods:**

A total of 539 patients were recruited into an observational, retrospective study when returning for a check-up between 3 and 36 months after an ischemic stroke. Patients’ modified Rankin Scale (mRS), dependency on caregivers, the Brandtstädter and Renner Coping questionnaire (with summary scores: Tenacity of Goal Pursuit (TGP) and Flexible Goal Adjustment (FGA) coping styles), EQ-5D-3 L and co-morbidities were evaluated.

**Results:**

In multivariable regression, greater disability (mRS) resulted in large utility losses, between 0.06 for mRS 1 to 0.65 for mRS 5 (*p* < 0.0001). Dependency on caregivers caused an additional dis-utility of 0.104 (*p* = 0.0006) which varied by mRS (0.044, 0.060, 0.083, 0.115, 0.150 and 0.173 for mRS 0–5). The effect of coping on utility varied by coping style, by the disability level of the patient and by his or her dependency on caregivers. FGA coping was associated with additional increases in utility (*p* < 0.0001) over and above the effect of disability and dependency, whereas TGA had no significant impact. FGA coping was associated with larger utility changes among more disabled patients (0.018 to 0.105 additional utility, for mRS 0 to mRS 5 respectively). Dependent patients had more to gain from FGA coping than patients who function independently of caregivers: utility gains were between 0.049 and 0.072 for moderate to high levels of FGA coping. In contrast, the same positive evolution in FGA coping resulted in 0.039 and 0.057 utility gain among independent patients. Finally, we found that important stroke risk factors and co-morbidities, such as diabetes and atrial fibrillation, were not predictors of EQ-5D utility in a multivariable setting.

**Conclusions:**

This study suggests that treatment strategies targeting flexible coping styles and decreasing dependency on caregivers may lead to significant gains in quality of life above and beyond treatment strategies that solely target disability.

## Introduction

Stroke is the most common cause of acquired disability worldwide. In stroke survivors, quality of life (QoL) is variably affected by multiple factors [1, 2]. These include patient characteristics (age, sex) [3, 4], stroke outcome (physical disability, repeat events) [5, 6], stroke-related complications (speech impediment, cognitive impairment, depression) [3, 7, 8], psychological factors (problem-solving versus emotion-focused coping style) [9–13] and changed aspects of daily living (place of residence, dependency on caregivers, mobility, returning to leisure activities) [3, 14, 15]. Each of these factors are known to affect QoL, however no study has investigated the additional or combined effect of these factors.

The modified Rankin Scale (mRS) is the most frequently used global outcome scale in ischemic stroke [16]. The mRS measures the degree of impairment in bodily functions and structures [17, 18]. Although it is inclined towards motor function, it also takes into account patient autonomy and activities of daily living [19]. Several large studies have documented the variation in EuroQol-5D (EQ-5D) utilities for different levels of the mRS [5, 6, 20], showing that *disability* was the major determinant of QoL after stroke. *Dependency on caregivers,* a frequent theme affecting QoL in stroke survivors, is closely linked to patients’ place of residence, i.e. dependent patients are mostly living in inpatient facilities where help from caregivers is nearby [8, 21, 22]. The link between dependency and disability is evident, and whilst it is documented that dependency on caregivers also negatively affects QoL, it is unclear whether there is an additional effect on QoL over and above the effect of disability. Finally, the impact of *coping style* on patients’ EQ-5D utility has also been documented. Coping is defined as the actions or emotions people develop to deal with stressful events. The literature on coping style demonstrates that stroke patients with flexible, problem-solving coping styles have higher QoL compared to patients with avoiding, resignation and denial coping styles, and have a lower probability of suffering from depression [9–13]. Coping strategies can be developed in a positive or negative way, and it has been shown that a good coping style can be learned [9, 23]. However, it is unclear what the specific impact of coping is on QoL in addition to dependency on caregivers and on the large and well-documented impact of disability.

This study set out to estimate the importance of a personal mind-set (coping ability), clinical factors (disability) and environmental factors (living in inpatient facilities) on stroke patients’ quality of life. The aim was to investigate whether there is an additional effect on the utility value of coping and of being dependent on caregivers, over and above the well-known effect of disability. The purpose of investigating factors that affect quality of life of stroke patients is to identify treatment strategies in addition to physical therapy that can enhance the quality of these patients’ lives.

## Methods

### BOI study design

In this observational, retrospective Burden of Illness (BOI) study, patients with ischemic stroke were recruited from ten hospitals in Belgium. Patient selection was on an “all comers” basis with stratification by mRS value and time since stroke (less versus more than 6 months). Patients were recruited when they returned to the hospital for a regular, scheduled in-person check-up visit after their index ischemic stroke. The timing of this visit varied between 3 to 36 months after stroke depending on the hospital’s follow-up policy. Patients with a major disability who had an mRS value of 4 or 5 were less able to attend the outpatient clinic and were recruited and interviewed (with their caregivers) by telephone or at their place of residence in the last year of the study. The hospitals were distributed throughout Belgium and included teaching and regional hospitals. Data was collected between September 2010 and May 2013. The ethics committees of the individual participating hospitals approved the study and informed consent was obtained from all patients or their caregivers. The resource use and cost data from the BOI study were published elsewhere [24].

### Outcome measures

At the time of patient’s check-up visit to the clinic, the physicians completed an mRS assessment. Physicians also completed data on co-morbidities (diabetes, previous stroke, transient ischemic attack) and medical risk factors (hypertension, hypercholesterolemia, smoking, atrial fibrillation). QoL data were collected using the EQ-5D-3 L questionnaire, which is a generic instrument to measure people’s health status and summarize patient’s QoL based on five domains: mobility, self-care, usual activities, pain/discomfort, and anxiety/depression [25–27]. Information on these five domains was combined in a single index, called utility value (see details in the statistical section below); it is this utility value that is used as the main outcome variable in all analyses. Patients gave information on their place of residence and whether they needed any mobility aids (wheelchair, walker) at the time of the follow-up visit. The definition of dependency in this study is based on a combination of patients’ place of residence, and whether or not patients need daily input from caregivers to perform their daily activities. Dependency on caregivers was therefore defined as living either in a nursing home, a rehabilitation home, or moving out of one’s own home to live with a family member, or living at one’s own home but with daily help from a caregiver. The patient was defined as being “independent” when he/she lived at his own home and did not need daily help from a caregiver. This definition is different from the “dependency” concept which is implicitly included in the mRS, where it is closely linked to mobility (unable to walk without assistance, being in a wheelchair, being bedridden) and the resulting need from assistance from others. The dependency concept in our study focused on patients who are dependent on the goodwill and time from caregivers to receive help, patients who may experience distress of having to move out of their own home in order to facilitate the assistance given, and the psychological weight of the burden they impose on family caregivers.

Coping was assessed using the assimilative-accommodative coping scale, consisting of two subscales: the *assimilative coping* or “tenacious goal pursuit” scale (TGP) which assesses whether patients can adjust the (new post-stroke) situation to their personal preferences; and the *accommodative coping* scale or “flexible goal adjustment” (FGA) which evaluates the opposite, namely whether patients can adjust their preferences according to the (new post-stroke) situation [28]. An example of the assimilative coping strategy would be to put more effort into occupational therapy session to be able to dress oneself again as this has been a personal goal (“When faced with obstacles, I usually double my efforts.”). An example of accommodative coping would be to accept the fact that one will never be able to walk without a walker anymore despite the wish to be capable of walking without assistance (“After a serious drawback, I soon turn to new tasks”). Patients often use these two strategies simultaneously, and this might evolve over time with assimilative coping playing a greater role in the acute phase after stroke, and accommodative coping being more prevalent in the chronic phase.

### Statistical analysis

The EQ-5D domain data were transformed into utility indexes using the published European algorithm [29]. The utility index takes a value between − 0.0743 and 1.000, with 0 representing death and 1 representing full health. This variable’s distribution is usually left skewed, bound by 1 and presents floor- and ceiling effects, and it was transformed to make it more amenable to statistical modelling by taking the complement (=1-Utility Index) to make the distribution right skewed and positive. The mRS is a 7-point scale with scores ranging from 0 (no symptoms at all) to 6 (death); the statistical analysis considered its ordinal nature. Dependency is a dummy variable and takes values 0 (independent) or 1 (dependent). The coping subscales, TGP and FGA, were calculated as the sum score of 15 items each, ranging from 0 to 60, with higher scores indicating a higher level of coping. These variables were treated as a continuous variable in regression analysis, or categorized into three mutually-exclusive clusters “low”, “medium” and “high” for use in figures.

The analysis was carried out in four steps; in first instance the univariable effects of time, the mRS, coping and dependency on utility are presented with boxplots. Secondly, it was investigated whether the coping strategies TGP and FGA were independent concepts from disability and dependency. Thirdly, a multivariable Generalized Linear Model (GLM) was estimated to disentangle and estimate the effects of disability, coping strategy and dependency on utility. A fully parameterized model was estimated including age, sex, co-morbidities, risk factors and socio-economic variables. Gradually, non-significant parameters were removed using the type 3 tests and adjusted Aikaike Information Criterion (AICC). The best fit to the data was a model with a normal distribution and log link, selected among the Identity and log link, Gamma and normal distributions. The selection was based on AICC as well as on the comparison of the range of the predicted utilities with the observed utilities. Testing for multicollinearity was performed with the variance inflation factor and with the Craemer’s V [30, 31]. Finally, each domain of the EQ-5D was examined using a cumulative logit model estimating the likelihood of scoring lower on each domain of the EQ-5D. No adjustment was made for multiple testing. All analyses were conducted in SAS 9.4.

## Results

### Step 1: Univariable effects on the EQ-5D utility of disability, dependency on caregivers, tenacity and flexibility

**Fig. 1:**
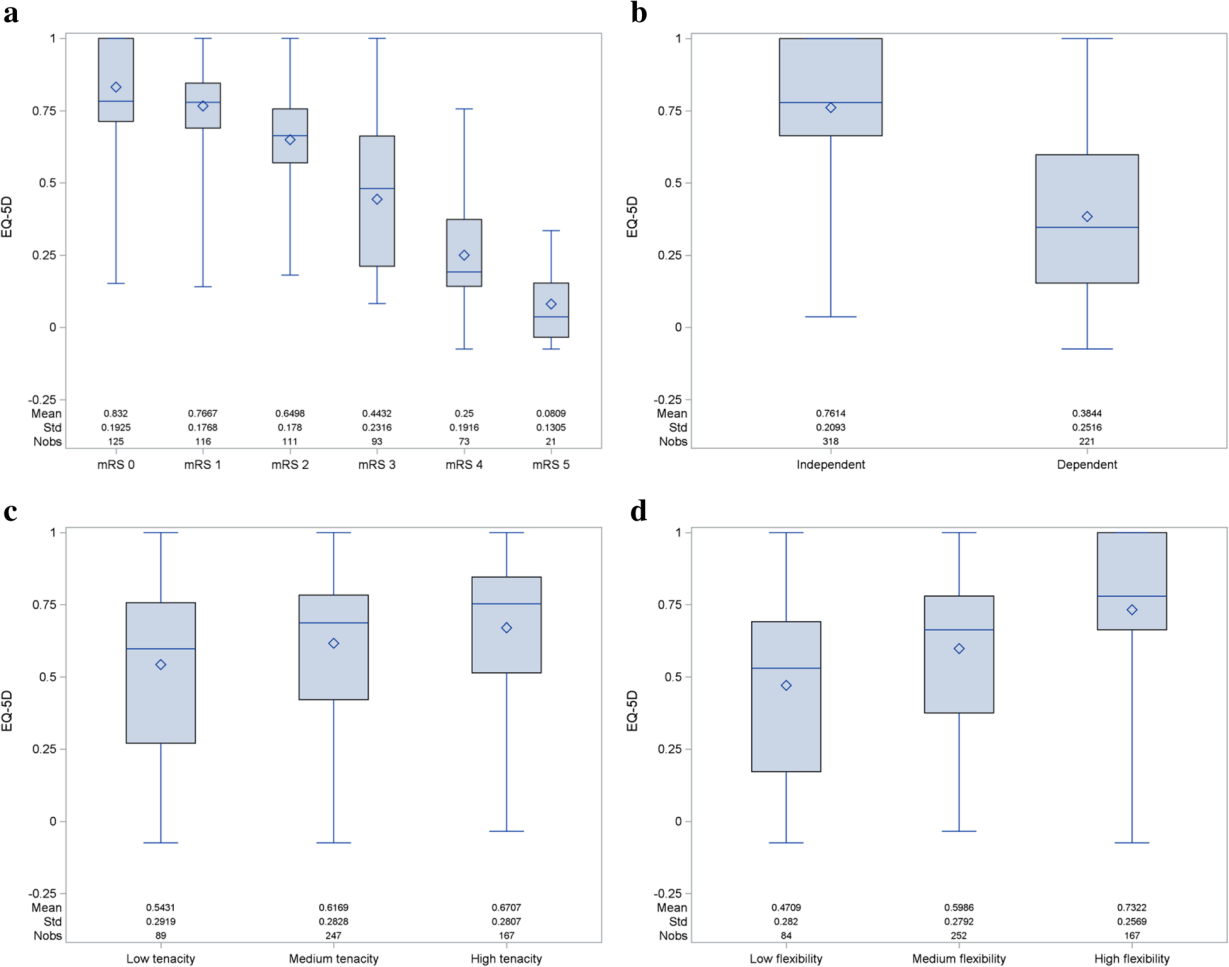
Univariable relationship between the EQ-5D utility and mRS (1**a**), dependency on caregivers (1**b**), tenacity (1**c**), and flexibility (1**d**)

A total of 539 patients who came for a scheduled follow-up visit after having an ischemic stroke were recruited in the BOI study dataset, patient characteristics are given in Table 1. Figures 1a-d depict boxplots showing the univariable relationship between the post-stroke EQ-5D utility, measured at a median of 6.1 months after stroke (IQR 3.7–14.8), and disability (mRS), dependency on caregivers, tenacity (TGP) and flexibility (FGA). Utility values differed significantly by the level of disability (*p* < 0.0001), with statistically significant differences found between each adjacent level of the mRS (all *p* < 0.01) (Fig. 1a). When testing for variation of the mRS-related utilities by age and sex, it was observed that females had a smaller utility change due to disability than males (*p* = 0.0025), and utility values were also generally lower in females. No significant interaction with age was found.

**Table 1:** Demographic and clinical characteristics

	mRS 0	mRS 1	mRS 2	mRS 3	mRS 4	mRS 5	All
N	125	116	111	93	73	21	539
% Female	35.8%	40.4%	36.7%	47.3%	48.0%	47.6%	41.1%
Age (mean, SD)	67.9 (12.3)	69.7 (11.5)	63.2 (14.6)	71.8 (11.4)	70.6 (13.2)	77.3 (9.6)	68.7 (12.9)
Hypertension	71.0%	76.8%	70.8%	70.7%	71.8%	90.5%	73.0%
Diabetes	19.0%	18.6%	15.7%	30.3%	18.8%	38.1%	20.9%
Atrial fibrillation	18.5%	22.3%	17.0%	30.3%	30.0%	45.0%	23.6%
Previous stroke	15.6%	6.5%	13.6%	17.4%	9.7%	33.3%	13.3%
History of TIA	19.4%	4.8%	3.4%	10.9%	12.9%	22.2%	10.4%
Months since diagnosis (mean, SD)	11.2 (11.5)	12.5 (17.1)	10.2 (10.1)	18.8 (25.5)	19.6 (72.1)	11.8 (12.1)	13.8 (30.8)

SD = standard deviation; TIA = Transient Ischemic Attack; mRS = modified Rankin Score

To investigate the effect of dependency (Fig. 1b), we compared the utility value of patients needing daily help from caregivers (*N* = 221, 41%) with patients who were able to live independently. The effect of dependency on caregivers on utility was to lower the post-stroke utility value in the BOI study by 0.377, from 0.761 to 0.384. The same effect was found when analysing these results by age and sex: no significant effect was found with higher age, but females had lower utility values regardless of dependency status, and also experienced a smaller utility decrement due to being dependent (0.358) than males (0.405; *p* = 0.020).

In Fig. 1c and d the relationship between utility and coping is shown. The coping variables were categorized into three levels, based on clustering analysis: TGP (low: 0–24, medium: 25–34, high: 35–60 score points) and FGA (low: 0–27, medium: 28–39, high: 40–60 score points). The average score points per TGP category were 19.54 (SD 4.65), 29.49 (SD 2.76) and 40.20 (SD 4.71), with an overall average of 31.28 (SD 8.21). The average score points per FGA category were 20.40 (SD 5.62), 34.13 (SD 3.22) and 44.49 (SD 3.62), with an overall average of 35.31 (SD 9.01). Patients with low levels of coping experienced lower EQ-5D utility values, and this can be observed for both the TGP and FGA strategies. The box plots show a stronger relationship between the EQ-5D utility and FGA compared to TGP. The utility increments for TGP were 0.0765 from low to medium tenacity and 0.0538 from medium to high tenacity. For FGA, the observed utility increases were 0.1277 for low to medium flexibility and 0.1336 for medium to high flexibility. Age and sex showed no significant effect (age: *p* = 0.079 for TGP and *p* = 0.45 for FGA, sex: *p* = 0.43 for TGP and *p* = 0.94 for FGA).

### Step 2: The independence of the concepts of tenacity and flexibility

**Fig. 2:**
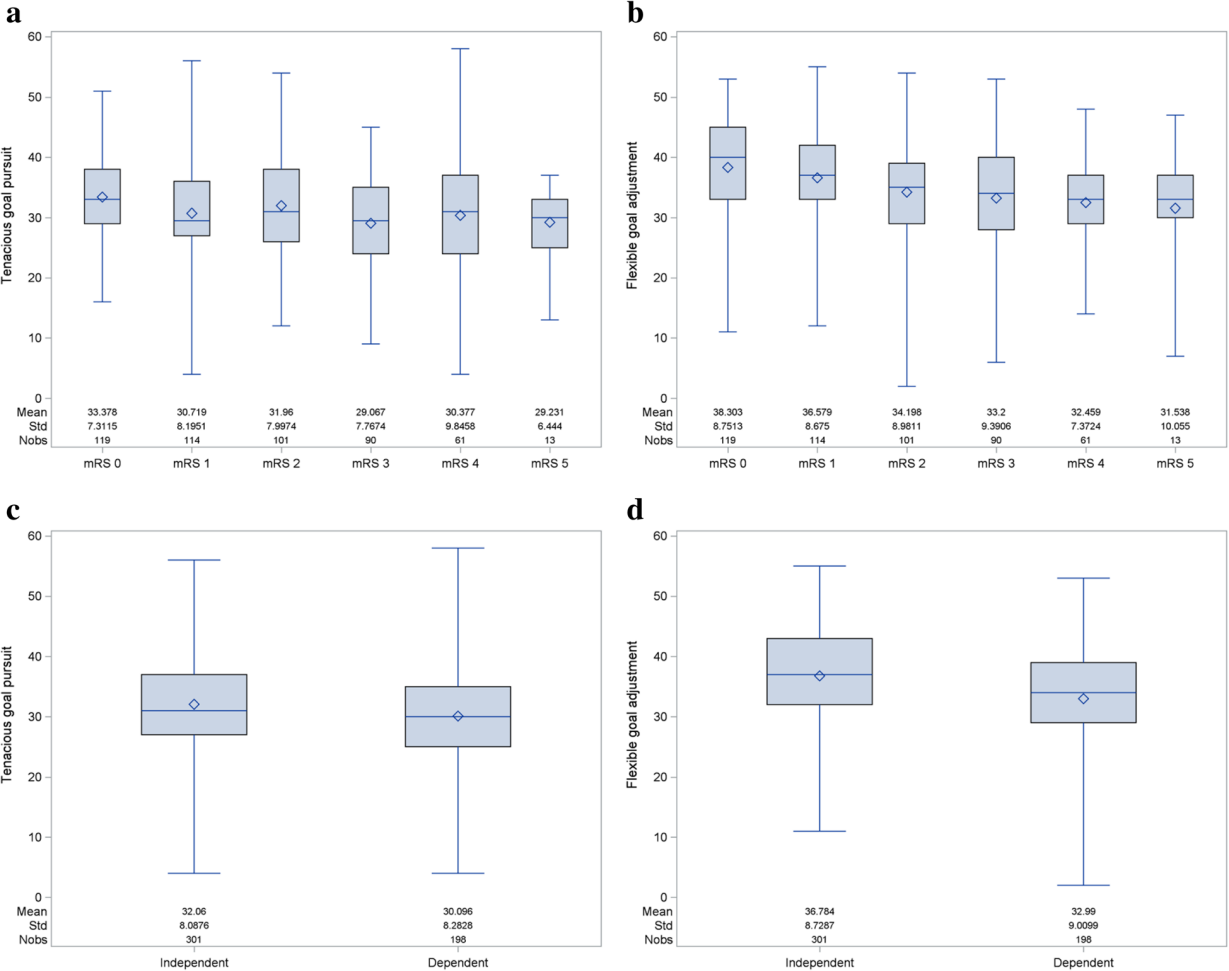
Association between TGP and mRS (2**a**), FGA and mRS (2**b**), TGP and dependency on caregivers (2**c**) and FGA and dependency on caregivers (2**d**)

In Figs. 2a-d, the associations between coping (TGP and FGA) and the mRS and dependency on caregivers is displayed. No strong relationship is present with any of the coping concepts: the full range of TGP and FGA score points is present at any level of the mRS or dependency on caregivers. Furthermore, when examining the relationship between tenacity and flexibility, it is apparent that they measure two very different concepts: tenacity explains only 6% of the variation in flexibility (regression-based approach, R-square = 0.06).

### Step 3: Multivariable analysis: The relationships between disability, dependency on caregivers and coping, and their combined effect on utility

The concepts of disability, dependency on caregivers and coping (TGP, FGA) all have an influence on QoL, however their effect might not be additive. Two integrated models were estimated: a simple model including only the mRS, dependency, TGP and FGA; and a more elaborate model adjusting these estimates for age and sex (Table 2). In this latter model, important risk factors (smoking, hypertension, hypercholesterolemia) and co-morbidities (diabetes, atrial fibrillation, previous stroke, TIA) associated with higher risk of stroke were explored but found not to be significant and therefore excluded. The mRS was the most significant contributor to explain the variance in utilities (*p* < 0.001), followed by FGA (*p* < 0.001) and dependency on caregivers (*p* = 0.0006). TGP was not significant in the simple model (*p* = 0.66), however a significant interaction was found with age in the elaborate model (*p* = 0.029). Age had the effect of increasing average utility values by 0.010 per 10 years of age (*p* = 0.017), ceteris paribus, whereas female sex reduced utility values by 0.028 compared to males (*p* = 0.0368).

**Table 2:** Results of the multivariable regression

	Simple model		Elaborate model	
	Parameter	*P*-value	Parameter	P-value
Intercept	−1.4004		−0.3828	
mRS 1	0.3089	<.0001	0.2890	<.0001
mRS 2	0.6403		0.6357	
mRS 3	0.9596		0.9529	
mRS 4	1.2250		1.2232	
mRS 5	1.3705		1.3874	
Dependency on caregivers	0.2298	0.0006	0.2180	0.0012
TGP	−0.0010	0.6642	−0.0305	0.0234
FGA	− 0.0096	<.0001	− 0.0095	<.0001
Age			−0.0146	0.017
Female			0.0842	0.0368
Age*TGP			0.0004	0.029

To generate predicted values based on this regression model: e.g. for a patient with mRS 3 who is dependent on daily help from others, has average tenacity (=TGP score 31.3), and high flexibility (=FGA score 44.6): utility value = 1-exp(−1.4004 + 0.9596 + 0.2298–0.0010*31.3–0.0096*44.6) = 0.488

The utilities based on calculations from the simple model are displayed in Table 3: utilities were calculated for different combinations of mRS, dependency, TGP and FGA. Each time, the effect of one parameter was varied whilst keeping the other variables at average levels. Based on these utilities, utility decrements were derived for the effect of the mRS, dependency on caregivers, TGP and FGA levels; these values are presented in Table 4.

**Table 3:** Average utility values by mRS, dependency, tenacity and flexibility scores, calculations based on the simple model

Effect of mRS:						
Dependency:	41% Dependent					
Tenacity:	Average: 31.3					
Flexibility:	Average: 35.3					
mRS 0	0.813					
mRS 1	0.745					
mRS 2	0.645					
mRS 3	0.511					
mRS 4	0.363					
mRS 5	0.263					
Effect of Flexible Goal Adjustment:						
Dependency:	Dependent	Dependent	Dependent	Independent	Independent	Independent
Tenacity:	Average: 31.3	Average: 31.3	Average: 31.3	Average: 31.3	Average: 31.3	Average: 31.3
Flexibility:	Low: 20.4	Med: 34.1	High: 44.6	Low: 20.4	Med: 34.1	High: 44.6
mRS 0	0.753	0.783	0.804	0.804	0.828	0.844
mRS 1	0.663	0.705	0.733	0.732	0.766	0.788
mRS 2	0.531	0.589	0.628	0.627	0.673	0.705
mRS 3	0.355	0.434	0.488	0.487	0.551	0.593
mRS 4	0.159	0.263	0.333	0.331	0.414	0.47
mRS 5	0.027	0.147	0.229	0.227	0.322	0.387
Effect of Tenacious Goal Pursuit:						
Dependency:	Dependent	Dependent	Dependent	Independent	Independent	Independent
Tenacity:	Low: 19.5	Med: 29.5	High: 40.2	Low: 19.5	Med: 29.5	High: 40.2
Flexibility:	Average: 35.3	Average: 35.3	Average: 35.3	Average: 35.3	Average: 35.3	Average: 35.3
mRS 0	0.783	0.785	0.788	0.828	0.829	0.831
mRS 1	0.705	0.708	0.711	0.765	0.768	0.77
mRS 2	0.589	0.593	0.597	0.673	0.677	0.68
mRS 3	0.434	0.44	0.446	0.55	0.555	0.56
mRS 4	0.262	0.27	0.277	0.414	0.42	0.426
mRS 5	0.147	0.155	0.164	0.322	0.329	0.336

**Table 4:** Utility differences due to dependency, flexibility and tenacity

	mRS > 0 vs. mRS = 0	Dependent vs. independent	Low vs. medium flexibility	Medium vs. high flexibility	Low vs. medium tenacity	Medium vs. high tenacity
mRS 0	ref	−0.044	−0.027	−0.018	−0.002	−0.002
mRS 1	−0.0677	−0.060	−0.036	−0.025	−0.003	−0.003
mRS 2	−0.1679	−0.083	−0.051	−0.034	−0.004	−0.004
mRS 3	−0.3015	−0.115	−0.070	−0.047	−0.005	− 0.005
mRS 4	−0.4500	− 0.150	−0.091	− 0.062	−0.006	− 0.007
mRS 5	−0.5498	− 0.173	−0.105	− 0.071	−0.007	− 0.008
Average Effect	−0.307	− 0.104	−0.063	− 0.043	−0.004	− 0.005
Note	With 41% patients dependent, TGP = 31.3 FGA = 35.3	With TGP = 31.3 and FGA = 35.3	With 41% of patients dependent and TGP = 31.3		With 41% of patients dependent and FGA = 35.3	

The mRS resulted in utility values varying between 0.813 and 0.263; the corresponding utility decrements were large: between − 0.068 and − 0.549 for mRS 1 to mRS 5 compared to mRS 0. The additional effect of dependency on caregivers was significant both statistically and clinically: over and above the effect of disability (mRS), dependency further reduced a patient’s utility after stroke on average by 0.104. This additional utility reduction due to daily dependency on caregivers varied by mRS and was − 0.044, − 0.060, − 0.083, − 0.115, − 0.150, − 0.173 for mRS 0 to 5, respectively. A gradient can be observed for the effect of being dependent on caregivers on utility values: this negative effect becomes larger as patients are more disabled, but even at moderate disability (e.g. mRS = 2), the effect of dependency (− 0.083) was found to be larger than the minimally important difference (MID) that is generally accepted for the EQ-5D utility values [32–35]. The effect of TGP on utility values was small in both models and below the threshold of the MID, as evidenced by the values in Table 4 (− 0002 to − 0.008).

The effect of FGA was significant across its whole range and at all levels of the mRS and of patients’ dependency status (Table 4). The potential utility increase from adopting a flexible coping style was largest among patients who are inflexible: coaching a patient from low (FGA =20.4) to medium flexibility (FGA = 34.1) resulted in 0.063 utility gain, versus 0.043 utility gain from medium (FGA = 34.1) to high flexibility (FGA = 44.6). Secondly, the effect of FGA was larger at higher mRS levels: the more disabled patients are, the more they can gain from a flexible attitude towards their goals. Estimated utility gains from learning a flexible coping style ranged from 0.018 for mRS 0 to 0.105 for mRS 5 (Table 4). Thirdly, it was found that the effect of FGA is also larger in patients who are dependent on caregivers compared to patients who are not. The utility increases among dependent patients resulting from low to medium, and medium to high flexibility were 0.072 and 0.049, whereas the same improvement in flexible coping style among independent patients resulted in 0.057 and 0.039 utility gains (calculations based on Table 3).

### Step 4: The impact of disability, dependency and coping on the individual EQ-5D domains

In Table 5 a set of odds ratios is presented for scoring one level lower (i.e. better) on each individual domain of the EQ-5D. Odds ratios lower than “1” indicate that the patient is more likely to experience problems in that domain with increasing values of that variable. Conversely, odds ratios higher than “1” indicate fewer problems with higher levels in this domain. Results show that patients with a higher mRS have more problems in each of the five domains, hence its strong impact on utility values. Furthermore, dependency on caregivers results in a higher likelihood of indicating problems with mobility, usual activities, and anxiety and depression. Surprisingly, no effect was found for dependency on problems with self-care. Likewise, patients with more flexible goal adjustment are likely to have fewer problems in most domains. Higher age was positively associated with mobility problems, and negatively associated with the likelihood of indicating problems with usual activities and pain. In addition, an association was found between female sex and the likelihood of problems with usual activities and pain. Finally, TGP was found not to have any impact on any domain of the EQ-5D.

**Table 5:** Odds Ratios for scoring one level lower on each EQ-5D domain

	Mobility	Self-care	Usual activities	Pain	Anxiety & depression
Age, per 10 additional years	**0.743**	0.889	**1.169**	**1.146**	1.076
Female	0.772	1.074	**0.555**	**0.639**	**0.510**
mRS	**0.316**	**0.175**	**0.264**	**0.651**	0.906
Dependent on others	**0.521**	0.775	**0.323**	0.834	**0.536**
Flexibility, per 10 additional point score	**1.359**	**1.429**	**1.590**	1.029	**2.277**
Tenacity, per 10 additional point score	1.113	1.111	1.010	1.164	1.166

Results in bold are statistically significant *p* < 0.05

## Discussion

### Comparison of our findings with published literature

The added value of our research is that it presents a multivariable analysis based on real-world data in which the effects of different determinants of QoL post-stroke are jointly estimated, and, moreover, that utility values are presented for use in economic evaluation. Our analysis demonstrated that dependency on caregivers plays an important role in influencing QoL, over and above the large effect of disability. Our study was able to quantify this additional effect (average reduction of 0.104 in utility) and showed a variation of the effect by level of physical disability. Furthermore, we found that coping style also has an important impact on utility, over and above the large effects of disability and dependency. The positive effect of coping varies by coping strategy (with FGA being associated with increases in utility, whereas TGA had no significant impact), by level of disability (higher disability being associated with higher utility increases due to FGA coping), and by dependency (dependent patients gain more utility from FGA coping than independent patients). We also found that important stroke risk factors and co-morbidities, such as diabetes and atrial fibrillation, were no significant predictors of QoL in a multivariable setting.

In contrast to our study, many previous publications have investigated the effect of determinants of utility in univariable analyses. Statistically significant utilities for each rank of mRS have been established based on large datasets [5, 6]. The importance of dependency on caregivers was also previously highlighted: patients who depended on others for activities of daily living (ADL) were found to report a consistently lower QoL score up to two years after stroke, compared to independent patients [15]. A recent long-term European study showed that for dependency on caregivers for ADL the following factors were each individually correlated with EQ-VAS scores up to 5 years after stroke: the burden imposed on the caregiver, disability, depression and anxiety [7]. This study, however, did not present a multivariable analysis combining all these factors. A recent Dutch study [10] found associations between the Brandtstädter and Renner coping questionnaire and the WHO QoL-BREF assessment. Accommodative coping (FGA) was found to be associated with higher QoL in the chronic phase after stroke compared to assimilative coping (TGA). The authors also observed that FGA was neither associated with physical health (pain, sleep, energy, mobility, work, ADL), nor with social relationships or with environment (e.g. freedom, place of residence, financial resources, availability of care, transport). FGA however was associated with psychological health (feelings, cognition, self-esteem, beliefs). A recently published Dutch study [9] followed patients for one year after stroke and concluded that psychological factors (including coping style and depression), place of residence and independence in ADL, were key in determining patients’ evolution of QoL. This study did however not account for patient’s disability and its large impact on QoL.

### Multi-disciplinary stroke care management

It is of importance to gain a better understanding of the determinants of QoL after stroke, in order to target stroke care to what really matters to patients. Our findings show that patients with very different post-stroke health profiles could all benefit from care targeted towards (1) reducing disability, (2) decreasing dependency on caregivers, and (3) training in effective coping strategies in order to positively influence their QoL. While current strategies mostly target reduction of disability and increasing independence, less formal attention is given to coping strategies.

A few observational studies and one randomized trial examined the efficacy of coping treatment strategies in stroke patients. A Dutch study [36, 37] conducted a 1-year randomized controlled trial (RCT) among stroke patients to investigate the effect of Problem Solving Therapy (PST) on coping skills and on QoL. PST is a psychological therapy to help patients identify problems, determine goals, generate solutions, select the best option and assist in the evaluation of the result. This study found that PST, on top of physical rehabilitation and Occupational Therapy (OT), was positively associated with coping skills and with the EQ-5D utility. A cross-sectional study in 166 stroke patients in two rehabilitation centers [38] found that high depression scores were significantly related to less positive problem-solving and emotional coping, and a lower EQ-5D utility score. The authors concluded that all stroke patients could benefit from training in coping skills, especially problem-solving skills. Another publication [39] discusses the results of a 1-year prospective study on the effect of PST on coping skills and QoL of stroke patients. Findings were similar to the other studies: PST was found to be associated with better FGA and TGP coping, and patients with higher levels of FGA coping also had higher EQ-5D utility scores. TGP on the other hand was not found to have an effect on utility. Other publications discuss the efficacy of psychotherapy with PST in different patient populations [40, 41]. These studies all conclude that psychological therapy with a problem-solving orientation will lead to increased coping skills among patients. Our study demonstrated that increased coping skills, in particular FGA, is associated with higher QoL in stroke patients, regardless of their level of physical disability or whether they are dependent on caregivers.

Whilst all acute therapies aim to reduce long-term disability, occupational therapy to further increase independence has not been studied as extensively. A recent Cochrane Review [42] based on 9 studies and 994 patients determined that post-stroke occupational therapy increased extended ADL (including mobility, household tasks and leisure activities), improved functional capability and reduced poor outcomes (i.e. death, deterioration in ADL, dependency in ADL or the need for institutional care). A few smaller clinical studies designed to investigate the efficacy of OT in gaining independence among stroke patients came to similar conclusions [43, 44]. Another systematic review [45] established that OT, with a focus on ADL-training, also improved basic ADL (dressing, feeding, hygiene) on top of extended ADL and the composite endpoint “poor outcome”. These findings show that OT leads to increased independence in stroke patients, which is confirmed by our study showing that reduced dependency on caregivers is correlated with higher QoL.

The clinical implications of these findings are that OT, with a focus on gaining independence, and psychotherapy, geared towards an accommodative, problem-solving coping style, given in addition to physiotherapy are the most promising way for gaining additional units of QoL. Using the combined care of physiotherapists, occupational therapists and mental health support is expected to maximize the potential gains in QoL after stroke.

### Study limitations

The main limitation of this research was the cross-sectional nature of the dataset: the study did not follow patients over time and was not able to capture the effect of changes in disability, dependency and coping on QoL. Furthermore, the wide range of time since stroke (between 3 and 36 months after stroke) may have influenced the results. A decrease in disease severity over time, due to higher mortality rates in more severe patients, is commonly observed in longitudinal studies (and was also found in the OxVASC study) [8]. This frequently results in a lower prevalence of severe patients in later time periods, causing an overestimation of utility values in later years after stroke. The BOI study was not biased due to the stratified design by mRS and by time since stroke. When we examined the patient characteristics over time, no different severity profile was found as time since stroke increased, and neither did the coping style. However, the different time-based samples may differ in unobserved characteristics that could affect outcome and QoL after stroke.

Furthermore, stroke may lead to major cognitive impairments and hence to the inability to participate in clinical studies. In the BOI study, patients were included in the study when they were coming back for a check-up; no exclusion criteria were defined based on patients’ cognitive function. The patients’ cognitive capabilities were not assessed, which is a limitation of our study. It is therefore not excluded that patients with cognitive problems responded themselves to our questions, possibly biasing results. In the BOI study, the caregiver (family member or nurse from the long-term facility) was allowed to assist their patient in filling in questionnaire. Seventy percent of patients filled in the EQ-5D themselves, whereas in 30% of cases EQ-5D was filled in by a caregiver or nurse (with the help from the patient). Published literature shows that there is a reasonable correspondence between patient and proxy ratings for EQ-5D [46], and that differences between the ratings are smaller than the minimally important difference [47]. The difference in assessment between patient and proxy is found to be moderate for the EQ-5D-3 L instrument, and the concordance is generally better for the utility index than for the individual domain data [48, 49]. We did not adjust our multivariable utility analysis for the type of EQ-5D responder (patient or caregiver) as this variable is strongly related to the mRS and to dependency on caregivers. Due to this correlation, we could not include this in the regression model without causing multicollinearity. 96% of the coping questionnaires were completed either by the patient or with explicit cooperation from the patient; in 4% of cases it was the caregiver who filled in the questionnaire without help from the patient. As the coping survey is very personal, informing about the patient’s mind-set and how he/she deals psychologically with a difficult situation, it is likely that the answers given by the caregiver biased the results. At first sight, results showed that the lack of patient cooperation led to lower TGP (28.2 vs. 31.3) and FGA (29.9 vs. 35.5) scores for questionnaires filled in without patient input compared to the scores given by the patients themselves. This could suggest that caregivers underestimate the coping efforts of the patient. Taking into account the patient’s age, gender and mRS, this difference in coping scores by responder to the questionnaire became negligible and insignificant (*p* = 0.49 for TGP and *p* = 0.09 for FGA). We also investigated the effect of the lack of patient input on the coping questions on the EQ-5D values (controlling for mRS, age and gender) and found that it had no effect (*p* = 0.59). It cannot be excluded that the caregivers underestimated the patients’ efforts in coping with their new post-stroke health state, but we did not consider this effect largely enough to exclude these data from our analysis.

The final limitation we would like to highlight, is the lack of a depression measurement in this study. The inclusion of depression scores, in relation to dependency and coping scores, would have been of added value for this multivariable analysis. It could have generated additional insights into the relative contribution of each factor on QoL after stroke, and it could have emphasised the role that psychotherapists may play in stroke rehabilitation.

## Conclusion

In conclusion, QoL after stroke is determined by disability, the level of dependency on caregivers and patients’ coping strategy. This study suggests that treatment strategies targeting flexible coping styles and decreasing dependency on caregivers may lead to significant gains in quality of life, above and beyond strategies that solely target disability. Therefore, a multi-disciplinary approach to stroke care, including physical rehabilitation for improving disability, occupational therapy for gaining independence in daily living and mental health support for training a flexible coping style and learning to apply this to all aspects of daily living is likely to result in highest QoL gains for stroke patients.

## Abbreviations

ADL: Activities of daily life
AICC: Aikaike information criterion
BOI: Burden of Inness in stroke observational study
EQ-5D: EuroQoL-5 Dimensions
Exp: Exponent
FGA: Flexible goal adjustment
GLM: Generalized linear model
IQR: Inter-quartile range
Med: Medium
MID: Minimally important difference
mRS: Modified rankin scale
OR: Odds ratio
OT: Occupational therapy
PST: Problem solving therapy
QoL: Quality of life
RCT: Randomized controlled trial
SD: Standard deviation
TGP: Tenacious goal pursuit
TIA: Transient ischemic attack
WHO: World Health Organisation
